# Expression signature and prognostic value of CREC gene family in human colorectal cancer

**DOI:** 10.1186/s12885-023-11303-5

**Published:** 2023-09-18

**Authors:** Junya Ning, Min Liu, Jing Shen, Deping Wang, Lijuan Gao, Huiyu Li, Jimin Cao

**Affiliations:** 1 Key Laboratory of Cellular Physiology at Shanxi Medical University, Ministry of Education, and the Department of Physiology, Shanxi Medical University, Taiyuan, 030001 China; 2 Department of General Surgery, Shanxi Bethune Hospital, Shanxi Academy of Medical Sciences, Tongji Shanxi Hospital, Third Hospital of Shanxi Medical University, Taiyuan, 030032 China; 3 Tongji Hospital, Tongji Medical College, Huazhong University of Science and Technology, Wuhan, 430030 China

**Keywords:** CREC family, Expression signature, Colorectal cancer, Prognosis, Bioinformatics

## Abstract

Colorectal cancer (CRC) is one of the malignant tumors with the highest morbidity and mortality and poor prognosis. The mammalian gene family of Cab45/reticulocalbin/ERC-45/calumenin (CREC) consists of RCN1, RCN2, RCN3, SDF4 and CALU. Although CREC family members have been associated with CRC, the expression pattern, prognostic value, and the role of CREC family in CRC remain unclear. In this study, the expression, survival and biological functions of CREC family in CRC were determined via bioinformatic datasets analysis and experimental verification on clinical CRC specimen. Bioinformatic analysis showed that the expression levels of most CREC family genes were higher in CRC tissues than in normal colorectal tissues. The qPCR and western blot results also revealed that the transcriptional and protein levels of CREC family were elevated in CRC tissues compared with adjacent tissues. Besides, CREC family was significantly correlated with advanced tumor stage and poor prognosis of CRC patients. The expression levels of CREC family had correlations with genomic mutation and methylation, and with the infiltration levels of CD4 + T cells, macrophages, neutrophils, and dendritic cells in the microenvironment of CRC. Functional networks enrichment analysis indicated that the genes of CREC family were essential factors for CRC metastasis. Collectively, these findings suggest that CREC family might be potential targets for the treatment of CRC and candidate prognostic markers for CRC patients.

**Supplementary Information:**

The online version contains supplementary material available at 10.1186/s12885-023-11303-5.

## Introduction

Colorectal cancer (CRC) represents approximately 10% of all cancers. CRC is the third most commonly diagnosed and second most fatal cancer globally. Approximately 9.4% of cancer-related deaths were due to CRC in 2020 [1]. CRC is largely asymptomatic until alarm features develop at advanced stages [2]. Clinically, more than half of colon cancer patients have undetectable small metastases before surgery [3]. Thus, it is an urgent need to identify new biomarkers for the early diagnosis and treatment to effectively enhance prognosis of CRC.

Mammalian Cab45/reticulocalbin/ERC-45/calumenin (CREC) protein family consists of reticulocalbin, ER Ca^2+^-binding protein of 55 kDa (ERC-55), reticulocalbin-3, Ca^2+^-binding protein of 45 kDa (Cab45), and calumenin, and these five proteins are encoded by five genes RCN1, RCN2, RCN3, SDF4, and CALU, respectively [4]. The CREC protein family contains multiple 'EF-hand' Ca^2+^-binding motifs, and participates in secretory pathway, signal transduction, as well as several disease processes [5, 6]. Although proteomic analysis showed that CREC family members are highly expressed in CRC, there was less experimental validation [7, 8]. The aberrant expression of CREC family members is associated with poor prognosis of several malignant tumors, including non-small cell lung cancer [9], bladder cancer [10], and glioma [11]. However, the prognostic value and the role of CREC family in colorectal cancer have yet to be fully elucidated and thus deserve extensive studies.

The present study aimed to identify the significance of CREC family in CRC progression. We comprehensively analyzed the expression patterns, prognostic values, immune cell infiltration and biological functions of CREC family in CRC using publicly accessible databases and further verified the bioinformatic results in human CRC cell lines and CRC tissues. We demonstrated the value of CREC family in CRC progression and the preliminary molecular mechanisms and suggested the possibility of CREC family as potential target for the treatment and prognosis evaluation of CRC.

## Materials and methods

### Oncomine analysis

The online gene expression array database Oncomine [12] (http://www.oncomine.org) was used to analyze the transcription levels of CREC family in different cancers. Student's *t* test was used to generate *p* values for the differences of the mRNA levels of CREC family members between clinical tumor specimen and normal control specimen. The critical *p* value was defined as 0.05 and the fold change was set as 1.5.

### GEPIA dataset analysis

The Gene Expression Profiling Interactive Analysis (GEPIA) web server is a valuable resource for gene expression analysis based on tumor and normal samples from the TCGA and the GTEx databases. GEPIA2 (http://gepia2.cancer-pku.cn/) provides the analysis of RNA sequencing expression data from 198,619 isoforms and 84 cancer subtypes as well as the analysis of a specific cancer subtype, and comparison between subtypes [13]. In our study, GEPIA was applied to verify the mRNA levels of CREC family and the prognostic value of CREC family in CRC.

### UALCAN

The UALCAN Database (http://ualcan.path.uab.edu) is an interactive portal for analyzing the RNA-seq and clinical data from TCGA [14]. In this study, UALCAN was used to analyze the mRNA levels of CREC family and the relationship between CREC gene expression and tumor stage in Colon adenocarcinoma.

### Kaplan–Meier plotter and ROC analysis

The Kaplan–Meier plotter [15] (https://kmplot.com/analysis/) is a web-based tool for analyzing the correlation between gene (mRNA, miRNA, protein) expression and survival. Here, Kaplan–Meier plotter was used to assess the prognostic value of CREC gene family through predicting the overall survival (OS) of CRC.

The Receiver Operating Characteristic (ROC) curve was established by “ROCR” package in R to further assess the sensitivity and specificity of the risk score for prognosis prediction. The area under ROC curve (AUC) was calculated. AUC > 0.6 was considered as a potential cancer biomarker for clinical utility. RNA-seq data and clinical data for CRC patients were obtained from the Gene Expression Omnibus (GEO) database GSE17538 in our analysis.

### Collection of human CRC tissues

The CRC tissues and paired normal adjacent colorectal tissues tested in the present study were collected from eleven CRC patients in Shanxi Bethune Hospital, Taiyuan, China (Table S1). The experiments on colorectal specimens from these patients were mainly designed to verify the reliability of bioinformatic analyses. The tissues harvested during surgeries were frozen in liquid nitrogen and stored at an ultra-low-temperature freezer for experiments of quantitative real-time PCR and western blot.

### Ethics statement

This study involving human participants were reviewed and approved by the Shanxi Bethune Hospital (Approval no.: YXLL-2019–051), and conducted according to the principles expressed in the Declaration of Helsinki of 1964 and later versions. All procedures followed were in accordance with the ethical standards of the responsible committee on human experimentation. All patients have signed informed consent.

### Quantitative real-time PCR

Quantitative real-time PCR (qPCR) was performed to examine the mRNA levels of CREC gene family both in CRC cell lines and CRC tissues, with normal cell line or normal colorectal tissues as controls. Briefly, total RNA was extracted using trizol reagent (Invitrogen, USA). The cDNA was synthesized with 2 μg RNA following the manufacturer’s instructions of the PrimeScript RT reagent kit (TAKARA, DRR047). The transcriptional levels of CREC family were analyzed by qPCR with SYBR Green PCR master Mix (Takara, DRR041A). Relative gene expression was normalized to the level of β-actin. Primers for β-actin was purchased from Sangon Biotech (Order NO. B661102). Primer sequences for qPCR are shown as follows:

RCN1 forward: 5'-GGATGGGTTTGTGGATCAGGATGAG-3',

RCN1 reverse: 5'-TCTTTGTCTAACTTCCCGTCCTTGTTC-3'.

RCN2 forward: 5'-CCTAATAATCAGGGCATTGCAC-3',

RCN2 reverse: 5'-CTTCAGAGAGCTTTTTGTCACC-3'.

RCN3 forward: 5'-GGGAACTTCCAGTACGACC-3',

RCN3 reverse: 5'-CTTTCCTCTGGGGTGAGTTG-3'.

SDF4 forward: 5'-GAGAGAGTAGCCAACAGGGAGGAG-3',

SDF4 reverse: 5'-CATCAAAGCCACCCAGGTCCTTG-3'.

CALU forward: 5'-TGGATTTACGAGGATGTAGAGC-3',

CALU reverse: 5'-TTTTAAACCTCCGCTCATCTCT-3'.

### Western blot

Briefly, equal amounts of protein were applied to SDS–polyacrylamide gel and electroblotted onto poly-vinylidene difluoride membranes. The membrane was blocked with 5% non-fat milk in TBST for 1 h at room temperature, and then incubated with primary antibodies overnight at 4 °C. At room temperature, the membrane was incubated with peroxidase-conjugated secondary antibodies for 1 h. And proteins were detected by a super ECL Prime detection kit (SEVEN, SW134-01).

### Immunohistochemistry

Tissue sections were deparaffinized in xylene and rehydrated with gradient ethanol. The tissue slides were placed in sodium citrate buffer (pH = 6.0) and bathed in water (94 − 99 °C) for 20 min to achieve antigen retrieval. Then 3% H_2_O_2_ was used to block endogenous peroxidase. Tissue sections were rinsed three times in phosphate buffer solution and then were incubated with primary antibody (CD206, 1:1000) at 4 °C for overnight. Subsequently, tissue sections were incubated with corresponding secondary antibody. The target protein was visualized with DAB chromogen. Three fields were obtained in each section. ImageJ was used to calculate the positive staining signals in each field.

### HPA dataset analysis

The Human Protein Atlas (HPA) is an open access database to map all the human proteins in cells, tissues, and organs using an integration of various omics technologies, including antibody-based imaging, mass spectrometry-based proteomics, transcriptomics, and systems biology [16]. In this study, immunohistochemical stains of CREC family were derived from HPA dataset.

### TFs-target and miRNAs-target of CREC gene family

Transcription factors (TFs) are proteins capable of binding DNA in a sequence-specific manner and regulating transcription of gene [17]. In this study, hTFtarget [18] (http://bioinfo.life.hust.edu.cn/hTFtarget), a comprehensive database for regulations of human TFs, was used to determine TFs regulating the expressions of CREC gene family. Besides, microRNAs (miRNAs) have been implicated in cell-fate determination and in various human diseases via inducing RNA-silencing and working as post-DNA transcription regulators [19]. Here, Starbase [19] (http://starbase.sysu.edu.cn/) and Targetscan [20] (http://www.targetscan.org/vert_72/) were used to predict the upstream miRNAs regulating the genes expression of CREC family.

### TIMER analysis

Tumor Immune Estimation Resource (TIMER) (https://cistrome.shinyapps.io/timer/) is a comprehensive resource for systematic analysis of immune infiltration across diverse cancer types based on 32 cancer types and 10,897 samples from TCGA [21]. TIMER was applied to determine the correlation between CREC family gene expression and the immune cell infiltration degree. Besides, TIMER was used to analyze the correlation of CREC family gene expressions.

### cBioPortal analysis with TCGA

The Cancer Genome Atlas (TCGA) has genomic sequence, expression, methylation, and copy number variation data on over 11,000 individuals who represent over 30 different types of cancer [22]. The cBioPortal for Cancer Genomics (http://cbioportal.org) provides a web resource for exploring, visualizing, and analyzing multidimensional cancer genomics data [23]. In this study, cBioPortal was used to analyze genetic and epigenetic alterations of CREC family from the colorectal adenocarcinoma (TCGA, Firehose Legacy) dataset with 640 samples.

### Protein–protein interaction (PPI) network analysis

The Search Tool for the Retrieval of Interacting Genes/Proteins (STRING) (https://string-db.org/) is an online tool to analyze the interaction relationship between proteins [24]. In this study, STRING was applied to obtain the top 50 co-expressed genes of CREC family and to construct the co-expression PPI network of CREC family.

### Gene Ontology (GO) and Kyoto Encyclopedia of Genes and Genomes (KEGG) pathway enrichment analysis

Metascape is a web-based portal designed to provide a comprehensive gene list annotation and analysis resource for experimental biologists. In terms of design features, Metascape combines functional enrichment, interactome analysis, gene annotation, and membership search to leverage over 40 independent knowledgebases within one integrated portal [25]. GO (http://geneontology.org) provides structured, computable knowledge regarding the functions of genes and gene products [26]. KEGG (http://www.kegg.jp/ or http://www.genome.jp/kegg/) is an encyclopedia of genes and genomes, including gene function and biological pathway information [27]. Here, the GO functions and KEGG pathways of CREC family genes and their top co-expressed 50 genes were obtained using Metascape (http://metascape.org).

### Statistical analysis

Data from at least three separate experiments were presented as mean ± standard error (SEM) and analyzed by *t*-test with SPSS 17.0 software (SPSS Inc., Chicago, IL, USA). Differences at *p* < 0.05 were considered statistically significant.

## Results

### Transcriptional and protein expression levels of CREC family in human CRC tissues

**Fig. 1:**
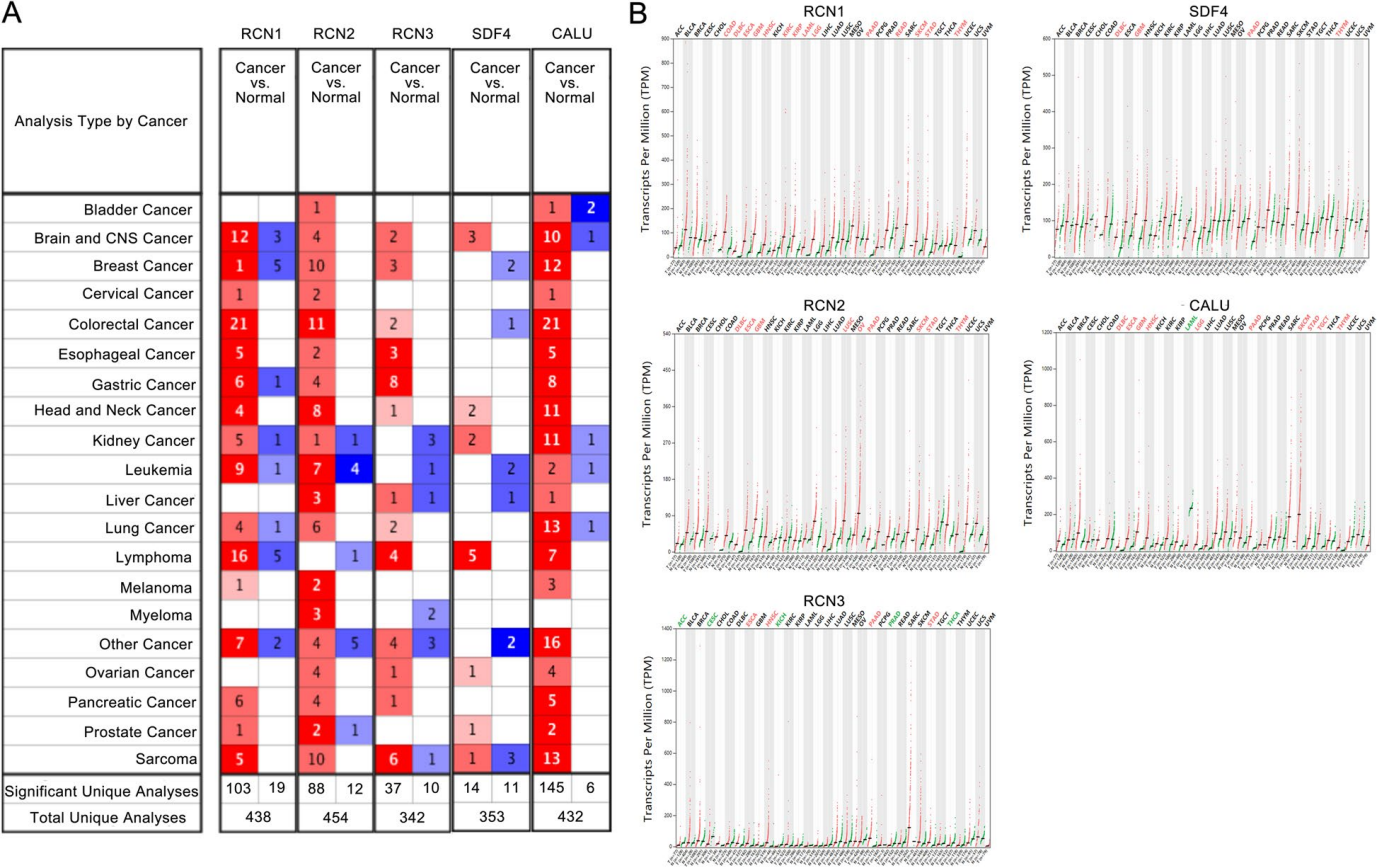
The mRNA expression patterns of RCN1, RCN2, RCN3, SDF4 and CALU in different types of human cancer. **A** Analysis results based on the Oncomine database. **B** Analysis results based on the GEPIA database

To compare the expression levels of CREC family in tumor tissues and corresponding normal tissues, we used Oncomine and GEPIA database to analyze the transcriptional levels of CREC family. Results from Oncomine revealed that the mRNA levels of CREC family were elevated in most tumors (Fig. 1A), which were roughly consistent with the results from GEPIA (Fig. 1B).

Results of various datasets from Oncomine showed that the mRNA levels of RCN1, RCN2, RCN3 and CALU were all significantly upregulated (fold change > 1.5) in CRC tissues versus normal tissues, while no significant result of SDF4 expression in CRC was found from Oncomine database.

Analysis on RCN1. Skrzypczak et al. [28] reported that RCN1 was overexpressed in colorectal carcinoma with a fold change of 2.048, in colon carcinoma epithelia with a fold change of 2.537, and in colon carcinoma with a fold change of 2.455. Sabates-Bellver et al. [29] showed that RCN1 was also overexpressed in colon adenoma with a fold change of 2.752, and in rectal adenoma with a fold change of 3.188. In The Cancer Genome Atlas, RCN1 was also overexpressed in rectosigmoid adenocarcinoma with a fold change of 2.616, in colon mucinous adenocarcinoma with a fold change of 2.361, in rectal mucinous adenocarcinoma with a fold change of 2.323, in cecum adenocarcinoma with a fold change of 2.124, in colon adenocarcinoma with a fold change of 2.197, and in rectal adenocarcinoma with a fold change of 2.052 (Table 1).

**Table 1:** Significant expression changes of CREC family at the transcription level between different types of CRC and normal colorectal tissues (from Oncomine Database)

**Genes**	**Types of CRC vs. normal colorectal tissue**	**Foldchange**	***p*-Value**	**t-Test**	**Source**
RCN1	Colorectal Carcinoma (36) vs. Normal (24)	2.048	5.71E-17	11.553	Skrzypczak [30]
	Rectosigmoid Adenocarcinoma (3) vs. Normal (22)	2.616	6.61E-8	12.153	TCGA
	Colon Mucinous Adenocarcinoma (22) vs. Normal (22)	2.361	8.36E-13	9.867	TCGA
	Rectal Mucinous Adenocarcinoma (6) vs. Normal (22)	2.323	1.47E-5	7.246	TCGA
	Cecum Adenocarcinoma (22) vs. Normal (22)	2.124	3.24E-10	8.022	TCGA
	Colon Adenocarcinoma (101) vs. Normal (22)	2.197	1.41E-13	10.849	TCGA
	Rectal Adenocarcinoma (60) vs. Normal (22)	2.052	3.00E-12	8.772	TCGA
	Colon Adenoma (25) vs. Normal (32)	2.752	1.69E-14	12.239	Sabates-Bellver [31]
	Rectal Adenoma (7) vs. Normal (32)	3.188	9.76E-6	9.777	Sabates-Bellver [31]
	Colon Carcinoma Epithelia (5) vs. Normal (10)	2.537	4.80E-8	10.858	Skrzypczak [30]
	Colon Carcinoma (5) vs. Normal (10)	2.455	1.42E-7	9.650	Skrzypczak [30]
RCN2	Rectal Adenoma (7) vs. Normal (32)	2.174	6.83E-14	14.305	Sabates-Bellver [31]
	Colon Adenoma (25) vs. Normal (32)	2.198	1.36E-14	10.940	Sabates-Bellver [31]
RCN3	Colorectal Carcinoma (36) vs. Normal (24)	1.526	1.56E-7	5.839	Skrzypczak [30]
SDF4	NA	NA	NA	NA	NA
CALU	Colorectal Carcinoma (36) vs. Normal (24)	2.106	8.99E-12	9.039	Skrzypczak [30]
	Colon Mucinous Adenocarcinoma (22) vs. Normal (22)	3.107	5.40E-13	10.190	TCGA
	Cecum Adenocarcinoma (22) vs. Normal (22)	2.583	1.05E-13	10.922	TCGA
	Rectal Adenocarcinoma (60) vs. Normal (22)	2.948	1.89E-15	13.852	TCGA
	Colon Adenocarcinoma (101) vs. Normal (22)	2.601	6.51E-14	12.377	TCGA
	Colon Carcinoma (5)vs. Normal (10)	2.604	2.86E-9	14.501	Skrzypczak [30]

Analysis on RCN2. In Sabates-Bellver’s dataset [29], RCN2 was found highly expressed in rectal adenoma with a fold change of 2.174, and in colon adenoma with a fold change of 2.198 versus normal samples.

Analysis on RCN3. Skrzypczak et al. [28] revealed that RCN3 was overexpressed in colorectal carcinoma (fold change = 1.526) versus normal samples.

Analysis on CALU. In Skrzypczak’s dataset [28], CALU was found highly expressed in colorectal carcinoma (fold change = 2.106) and in colon carcinoma (fold change = 2.604) versus normal samples. In The Cancer Genome Atlas data, higher expression of CALU was found in colon mucinous adenocarcinoma (fold change = 3.107), in cecum adenocarcinoma (fold change = 2.583), in rectal adenocarcinoma (fold change = 2.948), and in colon adenocarcinoma (fold change = 2.601) compared to normal samples (Table 1).

**Fig. 2:**
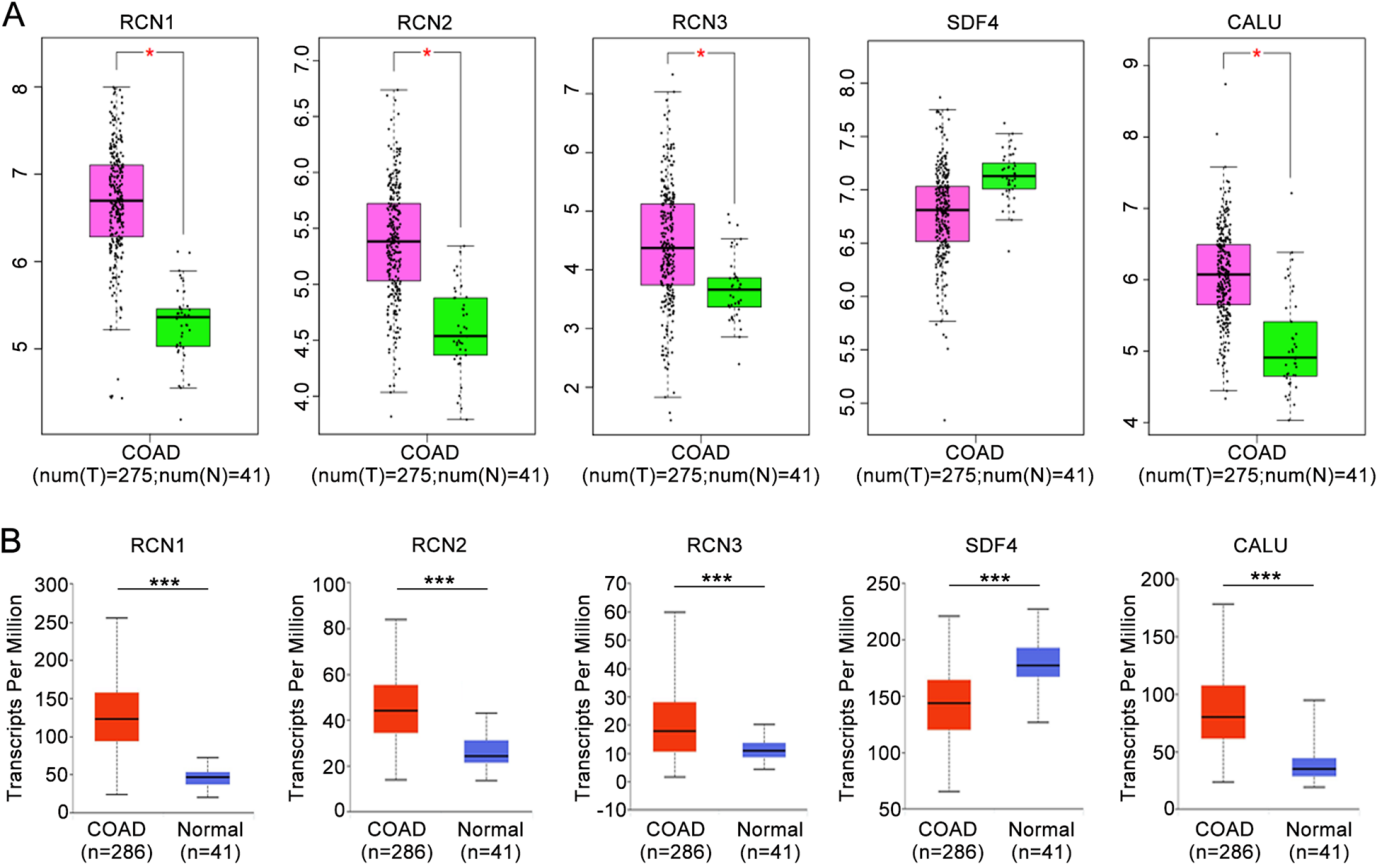
Expression level of CREC family in CRC tissues. **A** Analysis results based on the GEPIA database. **B** Analysis results based on the UALCAN Database

To further determine the relationship between CREC family and CRC, we used the GEPIA and UALCAN dataset to compare the mRNA expressions of CREC family between CRC and normal colorectal tissues. Results indicated that the transcriptional levels of RCN1, RCN2, RCN3 and CALU were significantly higher in CRC tissues than in normal colorectal tissues, while the transcriptional level of SDF4 was decreased in CRC tissues compared with normal colorectal tissues (Fig. 2A, B).

**Fig. 3:**
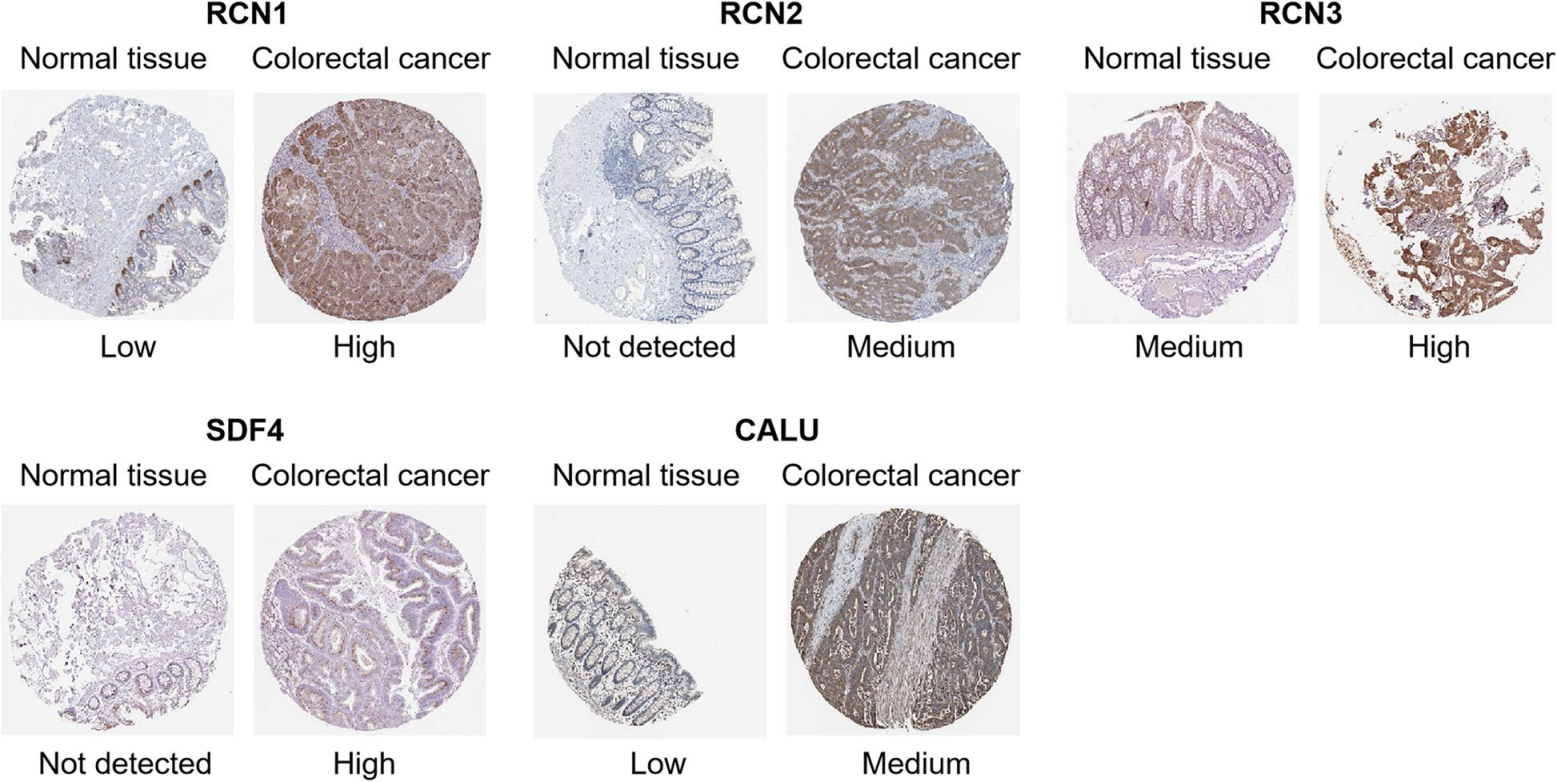
Representative immunohistochemical stains of distinct CREC family members in CRC tissues and adjacent normal tissues (derived from Human Protein Atlas)

We next evaluated the protein expression patterns of CREC family in CRC by the Human Protein Atlas (http://www.proteinatlas.org/). Results of immunohistochemical stains indicated that RCN2 and SDF4 protein were not detected in normal colorectal tissues, whereas medium expression of RCN2 and high expression of SDF4 were observed in CRC tissues. In addition, low expression of RCN1 and medium expression of RCN3 were observed in normal colorectal tissues, while RCN1 protein and RCN3 protein were highly expressed in CRC tissues. Besides, low expression of CALU was detected in normal colorectal tissues, whereas CALU showed medium expression level in CRC tissues (Fig. 3). These findings suggested that the protein expression levels of CREC family were higher in CRC tissues than in normal colorectal tissues.

**Fig. 4:**
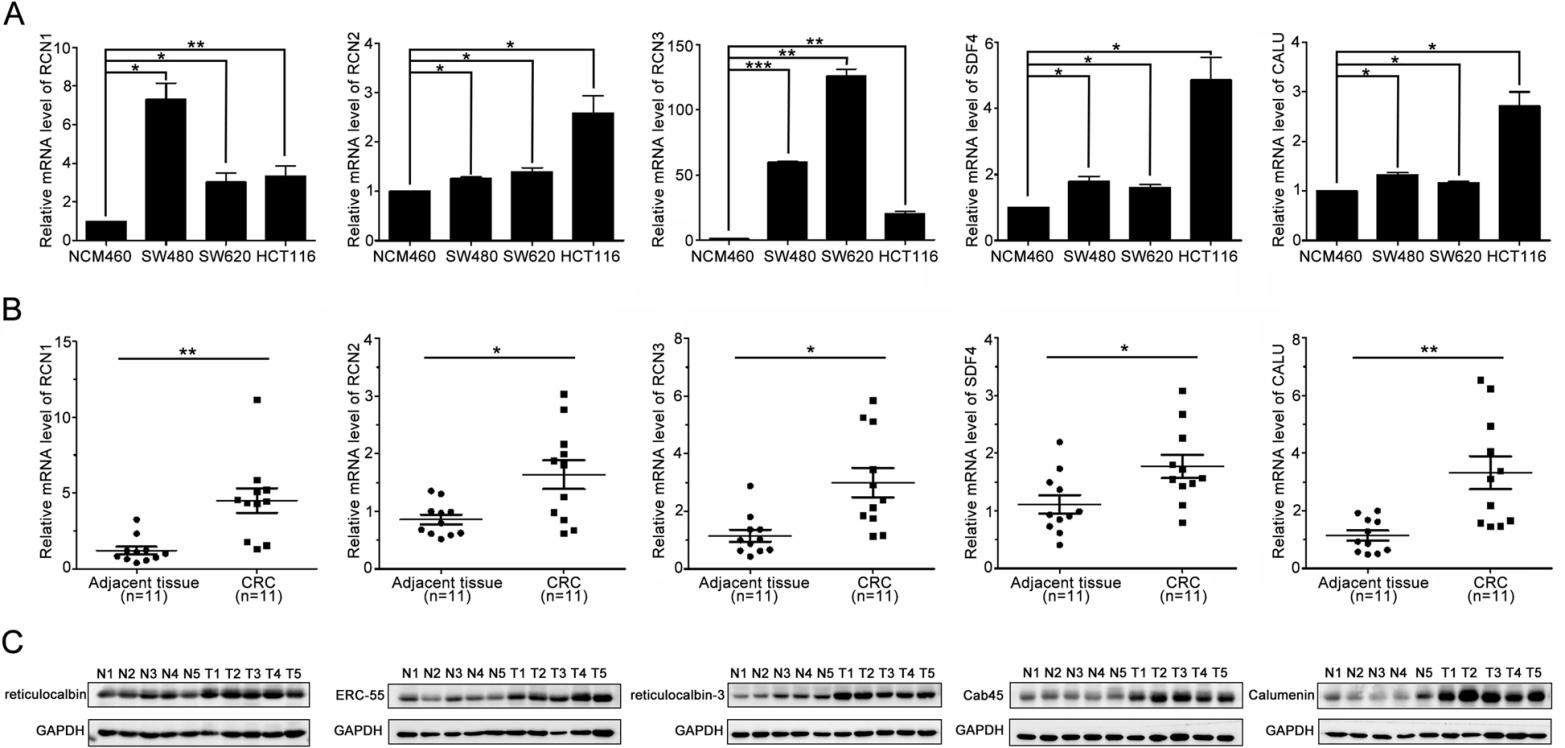
Elevated expressions of CREC gene family in CRC cell lines and tissues. **A** The mRNA levels of CREC family in normal colonic epithelial cell NCM460 and CRC cell lines (SW480, SW620, and HCT116) detected by qPCR (*n* = 3). **B** The mRNA levels of CREC family in CRC tissues (T1-T5) and normal adjacent tissues (N1-N5) from 11 CRC patients. **C** The protein levels of CREC family in CRC tissues and normal adjacent tissues from 11 CRC patients. Data were presented as the mean ± SEM, * *p* < 0.05, ** *p* < 0.01, *** *p* < 0.001

We further measured the expressions of CREC family in CRC cell lines and clinical CRC cases by using qPCR and western blot analysis. Results indicated that the mRNA levels of CREC family were elevated in CRC cell lines (SW480, SW620, and HCT116) compared with normal colonic epithelial cell NCM460 (Fig. 4A). The mRNA and protein levels of CREC family were significantly elevated in CRC tissues compared with adjacent tissues of 11 CRC patients (Fig. 4B, C).

### Transcription factors and microRNAs regulating CREC family expressions in CRC

**Fig. 5:**
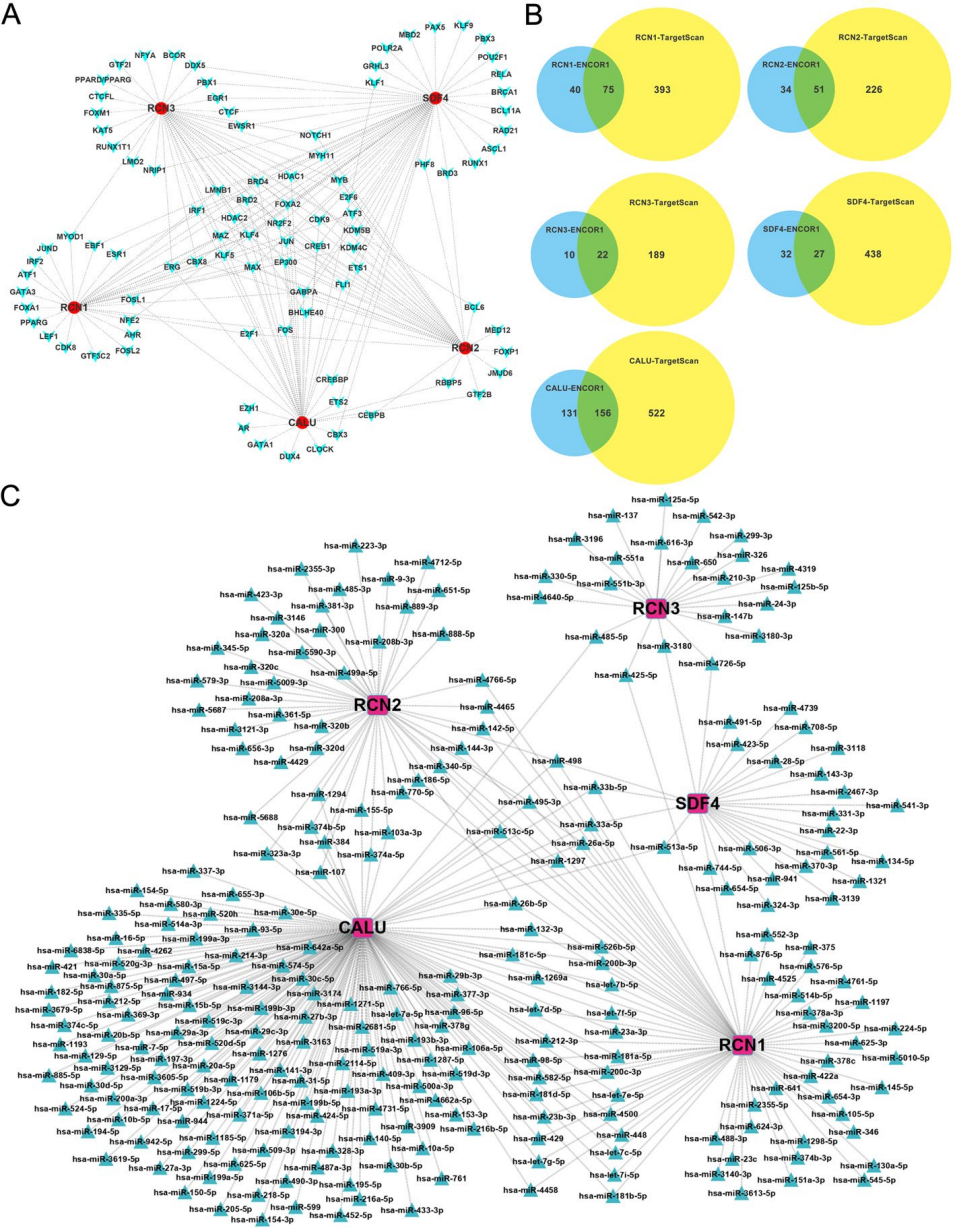
TFs-target and miRNAs-target that regulate CREC family. **A** TFs-target result (derived from hTFtarget). **B** miRNAs-target result of the intersection selected from Starbase and Targetscan databases (**C**) Detailed miRNAs-target result (derived from Starbase and Targetscan)

To better understand the genes expression regulation mechanism of CREC family in CRC, we used TF-target database (hTFtarget) and miRNA-target database (Starbase and Targetscan) to analyze the upstream TFs and miRNAs that regulate the expression of CREC gene family. We found 202 TFs (Fig. 5A) that regulate the expression of CREC gene family. Results of the intersection selected from Starbase and Targetscan databases revealed that the numbers of miRNAs that regulate RCN1, RCN2, RCN3, SDF4 and CALU were 75, 51, 22, 27 and 156, respectively (Fig. 5B). Overall, 329 miRNAs were found to regulate the expression of CREC gene family (Fig. 5C).

### Association of CREC family expression with the stage and prognosis of CRC

**Fig. 6:**
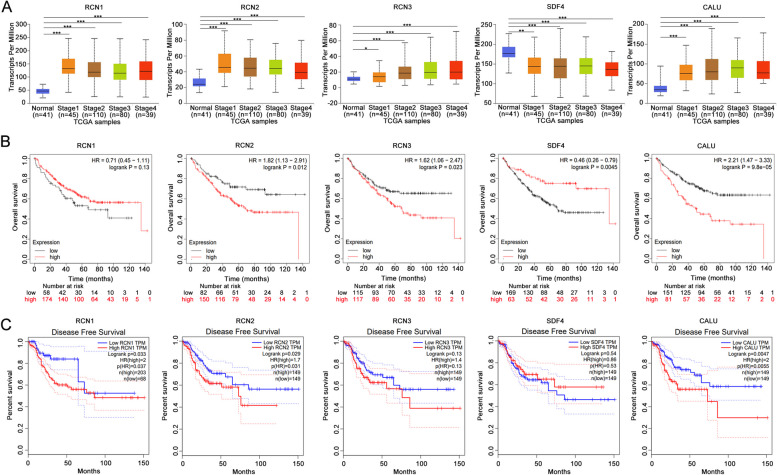
Significance of CREC expression in tumor stage and prognosis of CRC. **A** Correlation between the mRNA levels of CREC family and tumor stage in CRC patients (derived from UALCAN). **B**, **C** The prognostic value of mRNA levels of CREC family in CRC patients. Data were derived from Kaplan–Meier Plotter and GEPIA database

We analyzed the association of CREC gene family expression with the tumor stage of CRC using UALCAN. Results indicated that the expression of CREC genes was significantly associated with the stage and progression of CRC (Fig. 6A). In addition, Kaplan–Meier plotter was used to determine the correlation between the mRNA levels of CREC family and the survival of CRC patients. Results revealed that RCN2, RCN3, SDF4 and CALU were significantly associated with overall survival (OS) (*p* < 0.05) of CRC patients. CRC patients with high RCN2, RCN3 and CALU mRNA level were predicted to have poor OS, while CRC patients with low SDF4 mRNA level were predicted to have poor OS (Fig. 6B). Analysis based on GEPIA illustrated that increased mRNA levels of RCN1, RCN2 and CALU were significantly correlated with the disease-free survival (DFS) (*p* < 0.05) of CRC patients. CRC patients with high mRNA levels of RCN1, RCN2 and CALU were predicted to have poor DFS (Fig. 6C).

**Fig. 7:**
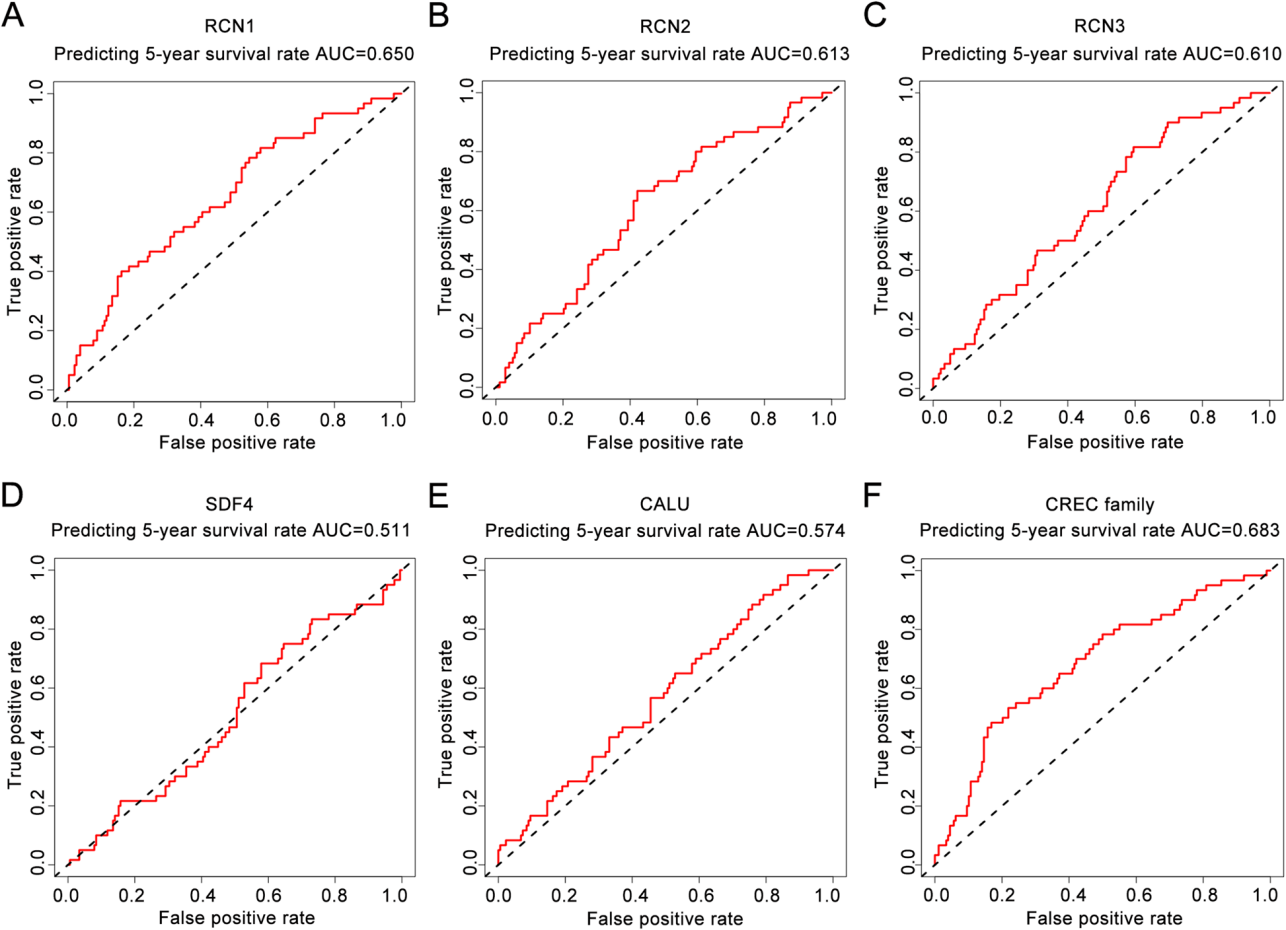
ROC curve of the risk score in inferior prognosis prediction of CREC family genes for 5-year overall survival in CRC

The ROC curve was established to further assess the prognostic performance of CREC family genes and combined value of these genes in CRC. We calculated the AUC for 5-year survival prediction. Results were as follows: RCN1 (AUC = 0.650), RCN2 (AUC = 0.613), RCN3 (AUC = 0.610), SDF4 (AUC = 0.511), CALU (AUC = 0.574), and combined analysis of CREC family genes (AUC = 0.683). RCN1, RCN2 and RCN3 possessed high prognostic values for CRC. A combination of CREC family genes yielded a better prognostic value for CRC (Fig. 7).

**Fig. 8:**
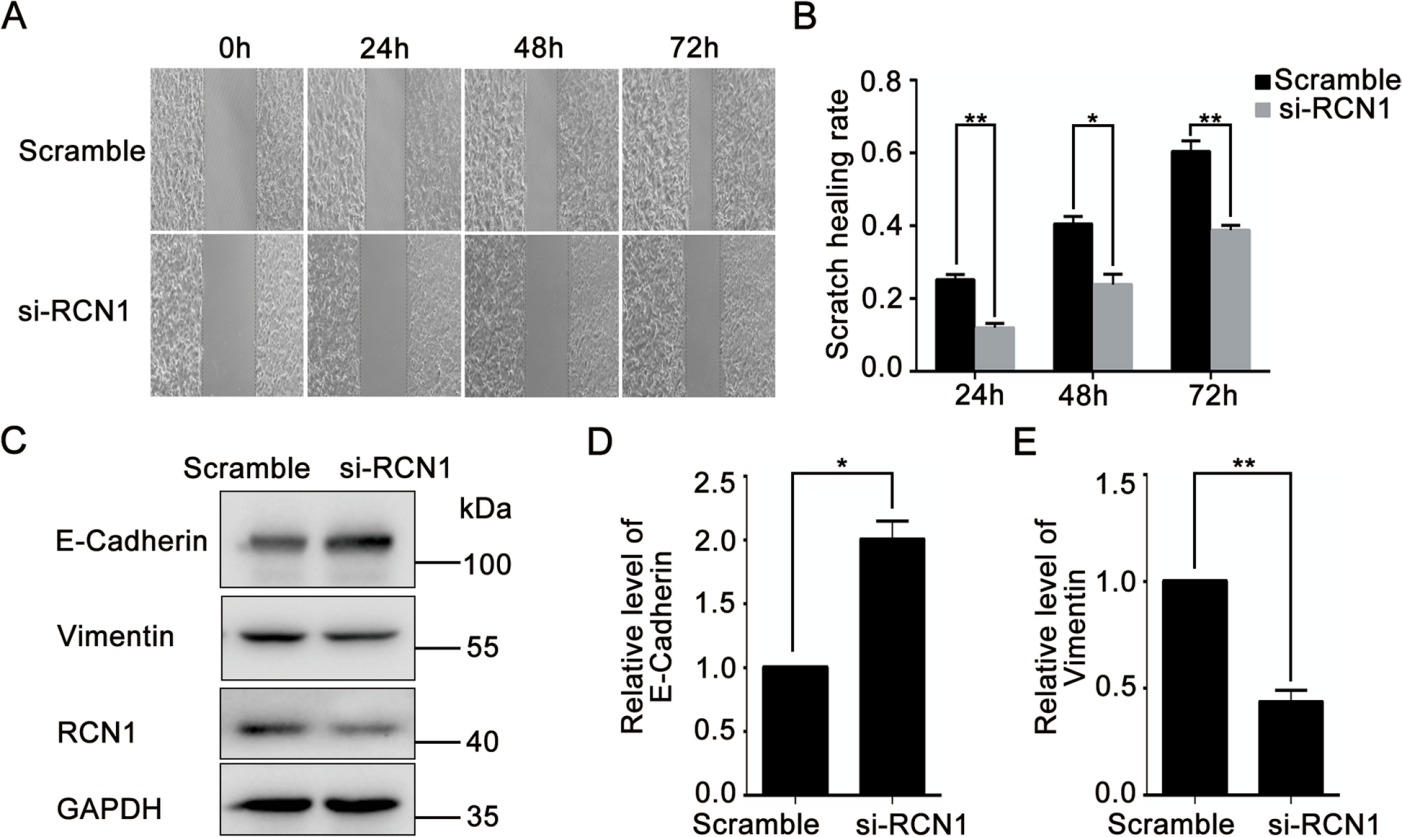
Knocking down RCN1 in SW480 cells effectively inhibited cell migration. **A**, **B** After knocking down RCN1 in SW480 cells, the scratch healing rate was measured. **C**-**E** Western blot analysis of E-Cadherin and Vimentin in SW480 cells with knockdown of RCN1. * *p* < 0.05, ** *p* < 0.01, *n* = 3

Migration and infiltration function play an essential role in tumor progression [32]. To explore whether RCN1 affected the migration of CRC cells, we conducted wound healing assay. Results showed that knocking down RCN1 was able to inhibit the scratch healing rate of SW480 cells (Fig. 8A, B). Then we analyzed the effect of RCN1 on the epithelial-mesenchymal transition (EMT) process of SW480 cells. The results indicated that knocking down RCN1 inhibited the EMT process of SW480 cells as evidenced by increased epithelial marker E-Cadherin and decreased mesenchymal marker Vimentin (Fig. 8C-E). These findings suggested that knocking down RCN1 effectively suppressed the migration of SW480 cells.

### Relation between CREC family expression and immune cell infiltration

**Fig. 9:**
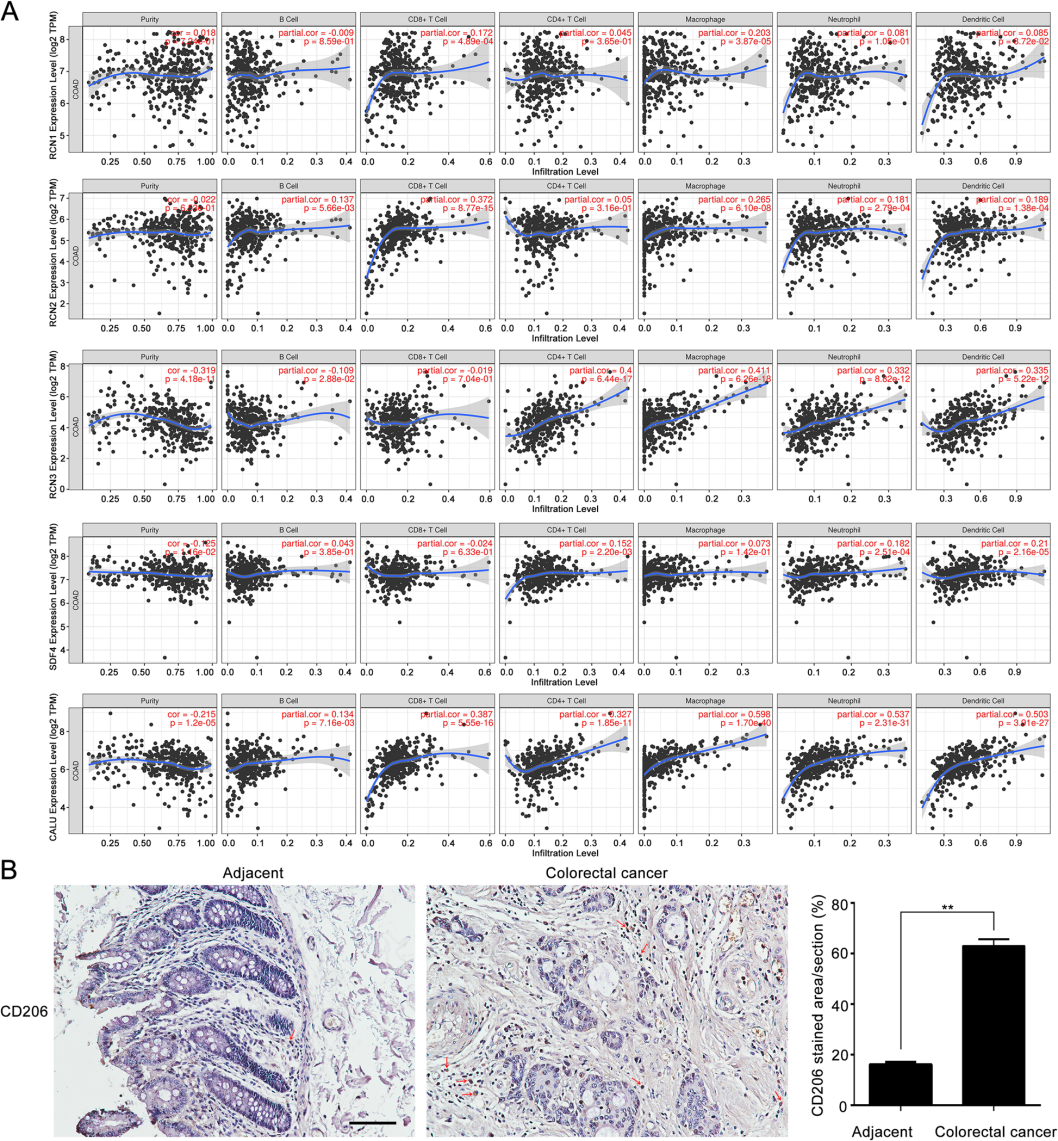
Correlation of CREC family expression with immune cell infiltration levels in CRC. **A** Analysis result derived from Timer. **B** Immunohistochemical images of CD206 from clinical CRC tissues and adjacent tissues included in this study. Scale bar: 100 μm. Data were presented as the mean ± SEM, ** *p* < 0.01

Given that infiltration degree of different immune cells plays an essential role in tumor progression and prognosis, we further explored the relationship of CREC family expressions with immune cell infiltration using TIMER database. Partial correlation (partial.cor) analysis indicated that RCN1 expression had a weak positive correlation with CD8 + T cell infiltration (*r* = 0.172, *p* < 0.05), while had a significantly positive correlation with macrophage infiltration (*r* = 0.203, *p* < 0.05). In addition, positive correlations were observed between RCN2 expression and the infiltrations of B cell (*r* = 0.137, *p* < 0.05), neutrophil (*r* = 0.181, *p* < 0.05), dendritic cell (*r* = 0.189, *p* < 0.05), CD8 + T cell (r = 0.372, *p* < 0.05), and macrophage (*r* = 0.265, *p* < 0.05). RCN3 (*r* =  − 0.319, *p* < 0.05) and CALU (*r* = – 0.215, *p* < 0.05) expressions had significantly negative correlations with tumor purity, while a weak negative correlation was observed between SDF4 expression and tumor purity (*r* =  − 0.125, *p* < 0.05). RCN3 expression showed significant positive association with the infiltration levels of CD4 + T cell (*r* = 0.400, *p* < 0.05), macrophage (*r* = 0.411, *p* < 0.05), neutrophil (*r* = 0.332, *p* < 0.05), and dendritic cell (*r* = 0.335, *p* < 0.05). Conversely, RCN3 expression had a negative correlation with B cell infiltration (*r* =  − 0.109, *p* < 0.05). SDF4 expression was positively correlated with the infiltrations of CD4 + T cell (*r* = 0.152, *p* < 0.05), neutrophil (*r* = 0.182, *p* < 0.05), and dendritic cell (*r* = 0.210, *p* < 0.05). CALU expression had positive correlations with the infiltration of various immune cells, including B cell (*r* = 0.134, *p* < 0.05), CD8 + T cell (*r* = 0.387, *p* < 0.05), CD4 + T cell (*r* = 0.327, *p* < 0.05), macrophage (*r* = 0.598, *p* < 0.05), neutrophil (*r* = 0.537, *p* < 0.05), and dendritic cell (*r* = 0.503, *p* < 0.05) (Fig. 9A).

Infiltration of inflammatory cells especially tumor-associated macrophages (TAMs) into the tumor tissue is an important feature of tumor microenvironment. To determine the clinical significance of TAMs in CRC, we examined the expression of TAMs markers (CD206) in tissues sections from 11 CRC cases. Results revealed that CD206 was highly expressed in CRC tissues compared with normal adjacent tissues (Fig. 9B).

### Genetic and epigenetic alterations of CREC family in CRC patients

**Fig. 10:**
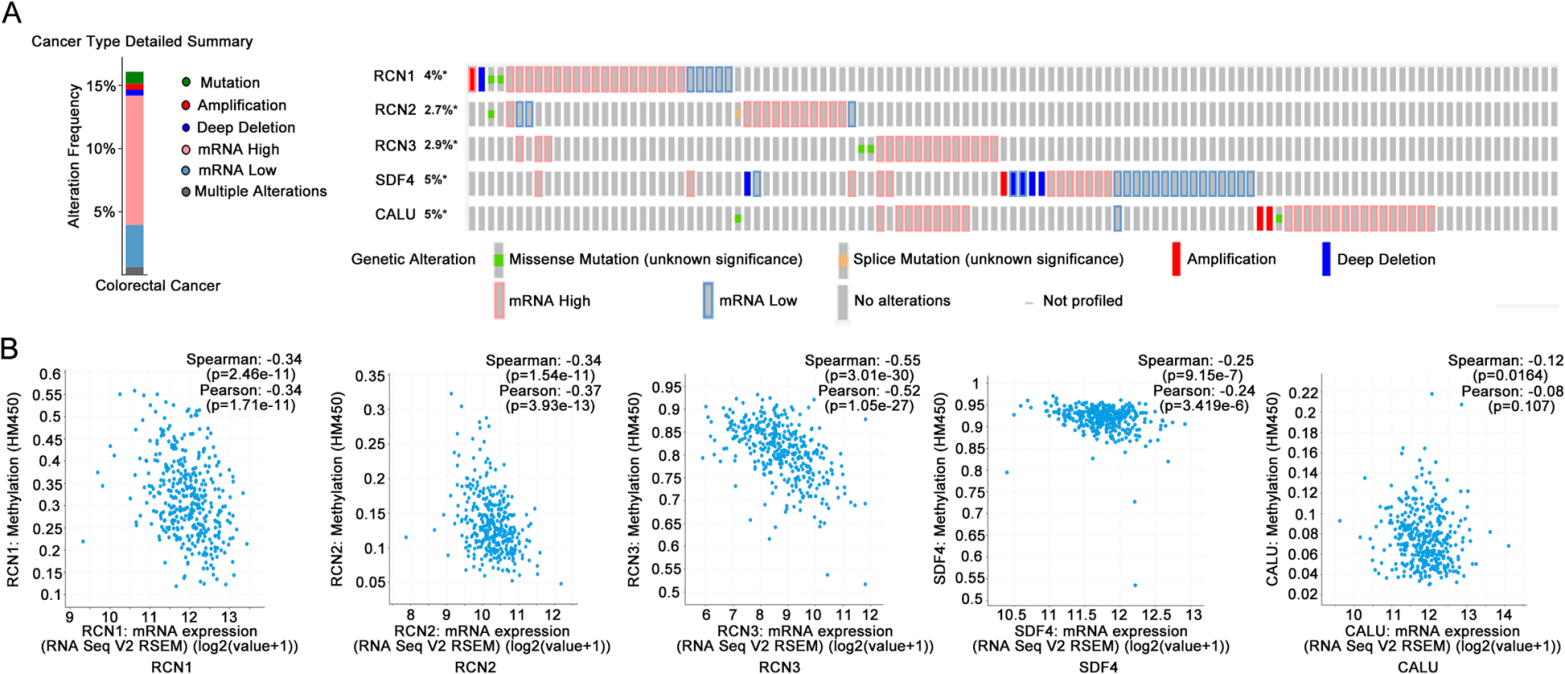
Analysis of CREC family gene mutation, expression and DNA methylation in CRC. **A** CREC family gene mutation analysis in CRC (derived from cBioPortal). **B** Correlation between gene expressions of CREC family and DNA methylation in CRC (derived from cBioPortal)

We further analyzed the alterations of CREC family in colorectal adenocarcinoma using the cBioPortal online tool. Results indicated that the genes of CREC family were altered in 630 samples of 636 patients with colorectal adenocarcinoma (32%). Results of detailed alteration in each gene revealed that gene alterations in RCN1 and CALU were amplification and deep deletion. Besides, RCN1 and CALU had two missense mutations respectively. RCN2 had one missense mutation and one splice mutation. RCN3 had two missense mutations. Amplification and deep deletions occurred in SDF4 (Fig. 10A).

DNA methylation is the methyl modification on the fifth carbon of cytosines (5-methylcytosine, 5mC) catalyzed by DNA methyltransferases (DNMTs). DNA methylation participates in maintaining the stability of genetic information, transcriptional inhibition and activation, X chromosome inactivation, reprogramming mammalian development and some diseases, including cancer [33]. In general, hypomethylation of the promoter regions of genes promotes their transcription [30, 34]. Lower DNA methylation levels are likely the contributed factors for the abnormal elevate of RCN3 in most cancers [31]. Here, the cBioPortal online tool was used to examine the relationship between expression pattern and DNA methylation level of CREC gene family. Data revealed that the expressions of CREC gene family showed a negative correlation with the level of DNA methylation (Fig. 10B), suggesting that DNA methylation plays an inhibitory role in the gene expressions of CREC family.

### Predicted functions and pathways of CREC family and their co-expressed genes in CRC

**Fig. 11:**
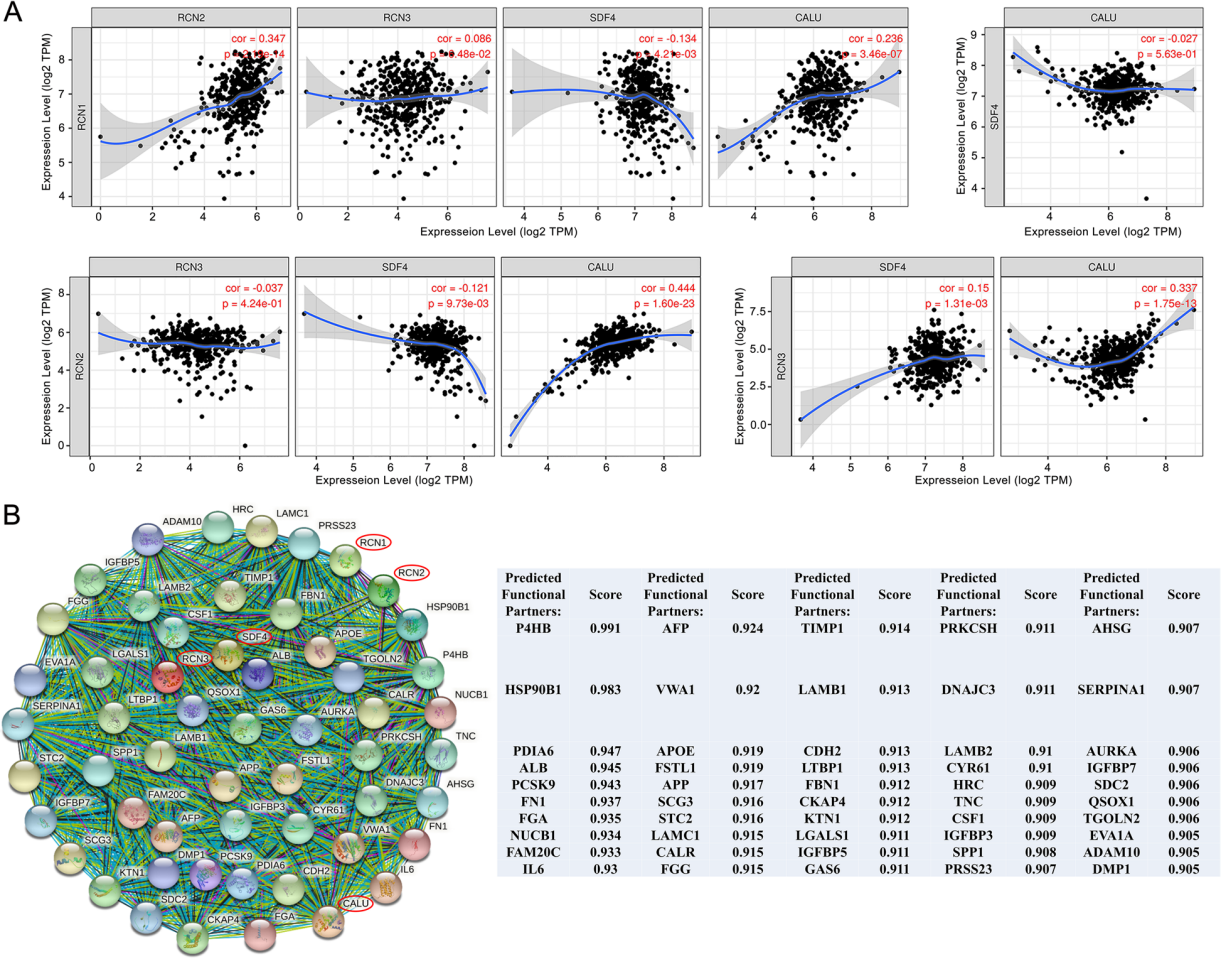
Relationships among CREC family and co-expressed genes of CREC family in CRC. **A** Potential correlations among RCN1, RCN2, RCN3, SDF4 and CALU. **B** The network for CREC family and the top 50 co-expression genes

Genes of the CREC family contain similar domains and may have partial overlapping functions. To explore the biological roles of CREC family in CRC, we first used TIMER to analyze the expression correlations of CREC gene family. Results indicated that RCN1 expression was significantly correlated with the expressions of RCN2 (*r* = 0.347, *p* < 0.05), SDF4 (*r* = − 0.134, *p* < 0.05) and CALU (*r* = 0.236, *p* < 0.05). RCN2 expression was significantly correlated with the expressions of SDF4 (*r* = − 0.121, *p* < 0.05) and CALU (*r* = 0.444, *p* < 0.05). Besides, RCN3 expression showed positive correlations with SDF4 (*r* = 0.15, *p* < 0.05) and CALU (*r* = 0.337, *p* < 0.05). However, the expressions of RCN1 and RCN3, RCN2 and RCN3, SDF4 and CALU had no significant correlation (Fig. 11A). These findings suggested that some genes of CREC family play a synergistic role in the progression of CRC.

**Fig. 12:**
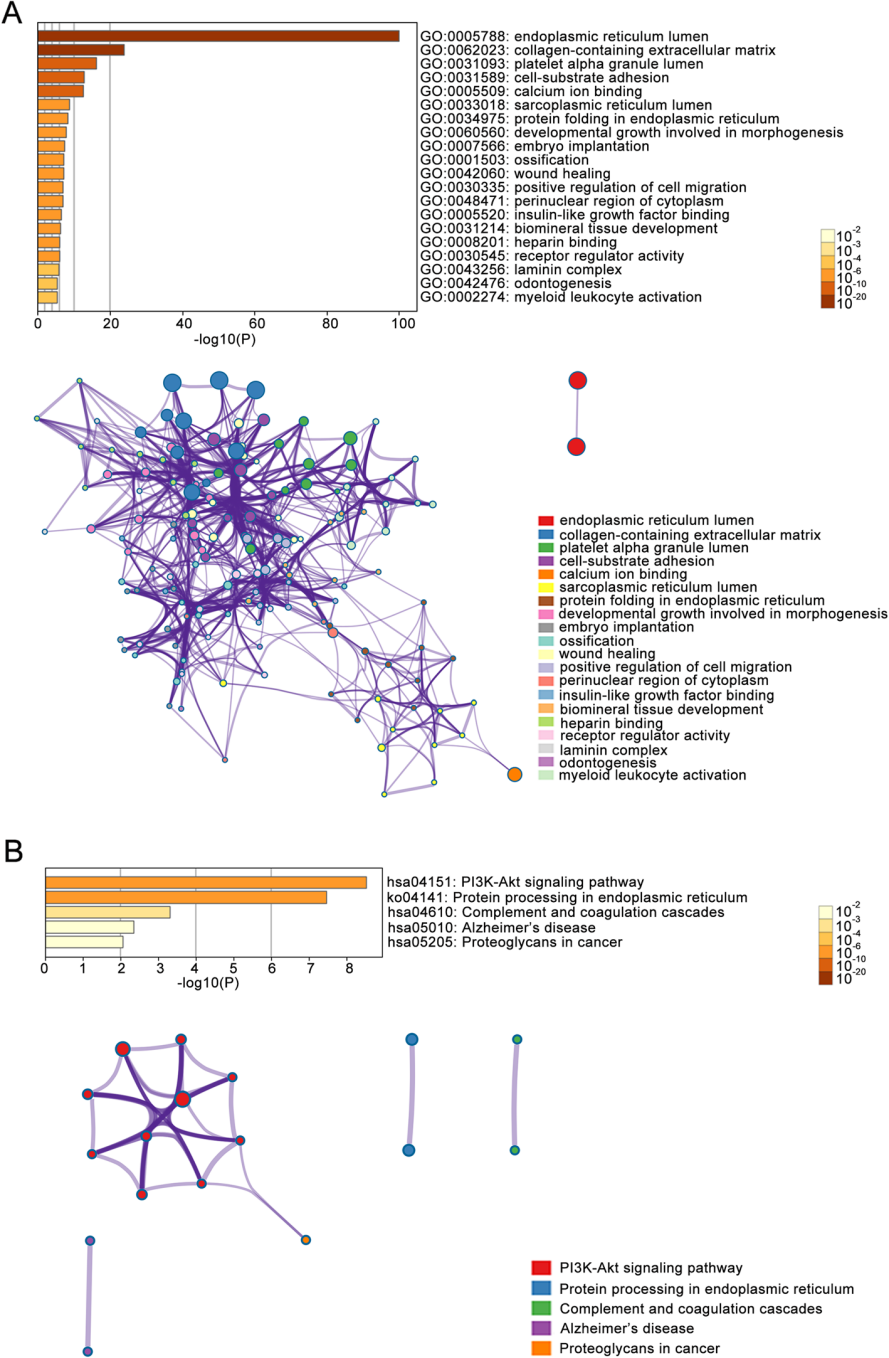
The Gene Ontology (GO) and Kyoto Encyclopedia of Genes and Genomes (KEGG) analyses on the functions of CREC family and the top 50 co-expression genes

We further analyzed the top 50 co-expressed genes of CREC family and their functional networks using STRING database (Fig. 11B). GO and KEGG analyses were conducted to determine the functions and related pathways enriched in the top 50 co-expression genes of CREC family. GO analysis showed that the functions of the top 50 co-expressed genes of CREC family were enriched in 10 biological processes (BP) including cell-substrate adhesion, protein folding in endoplasmic reticulum, developmental growth involved in morphogenesis, embryo implantation, ossification, wound healing, positive regulation of cell migration, biomineral tissue development, odontogenesis, and myeloid leukocyte activation. Besides, 6 cellular components (CC) identified by GO analysis encompassed endoplasmic reticulum lumen, collagen-containing extracellular matrix, platelet alpha granule lumen, sarcoplasmic reticulum lumen, perinuclear region of cytoplasm, as well as laminin complex. In addition, the molecular functions (MF) of the top 50 co-expression genes of CREC family were enriched in calcium ion binding, insulin-like growth factor binding, heparin binding, and receptor regulator activity (Fig. 12A, Tables S2-S4). KEGG pathway enrichment revealed that these genes were mainly enriched in the PI3K-Akt signaling pathway, protein processing in endoplasmic reticulum, complement and coagulation cascades, Alzheimer’s disease and proteoglycans in cancer (Fig. 12B), and the top two pathways were shown in Fig. S1. Collectively, CREC gene family members were involved in multiple cancer-related functions and pathways and were potential factors closely related to cancer invasion.

## Discussion

CREC family members have been implicated in cancer progression. Accumulating evidences suggest that RCN1 expression is significantly upregulated in various tumors, including CRC [9, 35]. Upregulation of RCN2 facilitates cell malignant behaviors and angiogenesis in cervical cancer and hepatocellular carcinoma [36, 37]. Overexpression of RCN3 is also associated with cancer progression [38]. The SDF4 gene, which contains seven exons and maps to 1p36.33 in chromosomes, encodes the 362-amino acid Cab45 protein. Alternative splicing of Cab45 mRNA results in three members: Cab45 of golgiosome (Cab45-G), Cab45 cytosolic (Cab45-C) and Cab45 secreted (Cab45-S). Cab45-G exhibits an increased expression in cell lines with higher metastatic potential and promotes cell migration in multiple types of cancer cells [39]. Cab45-S has been identified as a crucial modulator of tumor growth in cervical cancer cells [40]. Calumenin shows an increased expression in clinical tissue samples of colon tumors and acts as a novel putative biomarker of CRC [41, 42]. In the present study, the transcriptional levels of CREC family in CRC were systematically analyzed using Oncomine, GEPIA and UALCAN databases. Results indicated that RCN1, RCN2, RCN3 and CALU were all highly expressed in CRC compared with normal colorectal tissues. However, analysis based on GEPIA and UALCAN revealed that the transcriptional level of SDF4 was decreased in CRC, while analysis based on the Human Protein Atlas revealed that SDF4 protein expression significantly elevated in CRC. Our qPCR and western blot experimental data obtained from CRC tissues further showed that the expressions of CREC gene family were all significantly increased in CRC tissues compared with adjacent tissues. Hence, we speculated that the expression of SDF4 in CRC possessed tumor heterogeneity. An alternative potential explanation for differential SDF4 expression may be due to the difference of two subtypes (non-mucinous and mucinous type) of CRC. Besides, different expressions of SDF4 might also correlate with alternative splice variants. Given that the samples in the database are diverse, we will expand the sample size and include non-mucinous and mucinous type of CRC samples to investigate the above possibilities in our future study.

Genes of CREC family are also closely related to cell migration and cancer prognosis. RCN1 expression correlates with lymph node metastasis, migration and invasion of cancer cells [43]. Besides, RCN1 is highly expressed in invasive breast cancer cell and colorectal cancer cell, suggesting that RCN1 is implicated in tumor cell invasiveness. We further found that silencing of RCN1 was able to suppress CRC cell migration (Fig. 8). Moreover, overexpression of RCN1 correlates with poor prognosis of non-small cell lung cancer and glioblastoma [44–46]. Increased RCN2 level plays a vital role in hepatocellular carcinoma (HCC) proliferation, invasion and migration and predicted poor prognosis in HCC patients [47]. RCN2 also enhances the proliferation and invasion of colorectal cancer cells [48]. Cox's proportional hazards analysis showed that high RCN2 expression was an independent prognostic marker of poor outcome in colorectal cancer. Knockdown of RCN2 inhibited colorectal cancer cell proliferation both in vitro and in vivo [49]. RCN3 is considered a fibroblast-specific biomarker of poorer prognosis of CRC [50]. Upregulation of Cab45-S favors tumor growth and seems correlated with the cervical carcinoma grade [40]. High expression levels of Cab45 are correlated with cancer progression and metastasis [51] Silencing of Cab45-G remarkably inhibited cancer cell migration [39]. Increasing evidence indicate that overexpression of CALU promotes cancer cell growth, migration, invasion and metastasis [52, 53]. Knockdown of calumenin suppressed invasiveness of lung cancer cells [53]. Here, we reported that genes of CREC family were significantly related to the tumor stage and prognosis of CRC. Furthermore, combination of CREC family genes performed as a better prognostic marker for CRC. Thus, our findings suggested that CREC family were key factors in CRC progression and acted as candidate biomarkers for CRC prognosis. In addition, the functional networks of the top 50 co-expressed genes of CREC family in CRC mainly enriched in cell migration, also suggesting that CREC family played an important role in tumor metastasis. In the next study, we will further elucidate the specific mechanism of each member in regulating CRC metastasis and progression, thus providing new targets for CRC therapy.

Tumor microenvironment is mainly composed of cancer-associated fibroblasts (CAFs), immunosuppressive immune cells (regulatory T cells, M2 macrophages, myeloid-derived suppressor cells), extracellular matrix, a variety of growth factors, and inflammatory factors [54]. The infiltration degree of different immune cells is highly correlated with tumor survival and progression [55]. TAMs represent one of the main tumor-infiltrating immune cell types, mostly with the phenotype of M2 macrophages. TAMs promote tumor metastasis and are closely related to poor prognosis [56]. Here, we found that CD206 (a marker of M2 macrophage) in CRC tissues was highly expressed, indicating increased M2 macrophage infiltration in CRC. Therefore, we speculate that RCN1, RCN2, RCN3, and CALU are associated with poor prognosis for CRC by regulating macrophage infiltration. And this hypothesis will be further verified in future studies.

## Conclusion

We systematically analyzed the expression, prognostic value, and molecular biological functions of CREC family in CRC. Results indicate that the expressions of RCN1, RCN2, RCN3, and CALU are significantly higher in CRC tissues than in normal adjacent tissues, whereas the expression of SDF4 is controversial. The expression of CREC family is significantly related to CRC progression. Combination of CREC family genes is a potential prognostic marker for CRC. Furthermore, CREC family may play an important role in CRC oncogenesis and invasion. Our findings suggest that genes of CREC family might be potential therapeutic targets for CRC.

### Supplementary Information

**Additional file 1:** **Table S1.** Characteristics of the patients diagnosed with colorectal cancer. **Table S2.** Biological processes (BP) of GO analysis on the top 50 co-expressed genes of CREC family. **Table S3.** Cellular components (CC) of GO analysis on the top 50 co-expressed genes of CREC family. **Table S4.** Molecular Function (MF) of GO analysis on the top 50 co-expressed genes of CREC family. **Fig. S1.** PI3K-Akt signaling pathway and protein processing in endoplasmic reticulum of CRC. (A) PI3K-Akt signaling pathway and (B) protein processing in endoplasmic reticulum regulated by CREC family and the top 50 co-expression genes in CRC.

## Data Availability

Oncomine (http://www.oncomine.org), GEPIA2 (http://gepia2.cancer-pku.cn/), UALCAN Database (http://ualcan.path.uab.edu), Kaplan–Meier plotter (https://kmplot.com/analysis/), Cell Culture Bank of the Chinese Academy of Sciences (http://www.cellbank.org.cn/), hTFtarget (http://bioinfo.life.hust.edu.cn/hTFtarget), Starbase(http://starbase.sysu.edu.cn/), Targetscan (http://www.targetscan.org/vert_72/), Tumor Immune Estimation Resource (TIMER, https://cistrome.shinyapps.io/timer/), The cBio Cancer Genomics Portal (cBioPortal, https:// www. cbiop ortal.org/), STRING (https://string-db.org/), GO(http://geneontology.org), KEGG(http://www.kegg.jp/ or http://www.genome.jp/kegg/), Metascape (http://metascape.org), the Human Protein Atlas (http://www.proteinatlas.org/).
