# Supplementary material for: Expression signature and prognostic value of CREC gene family in human colorectal cancer
Source: BMC Cancer. 2023 Sep 18;23:878. doi: 10.1186/s12885-023-11303-5 (PMC10506189; doi:10.1186/s12885-023-11303-5)
Supplement: Supplementary file 1 — Additional file 1: Table S1. Characteristics of the patients diagnosed with colorectal cancer. Table S2. Biological processes (BP) of GO analysis on the top 50 co-expressed genes of CREC family. Table S3. Cellular components (CC) of GO analysis on the top 50 co-expressed genes of CREC family. Table S4. Molecular Function (MF) of GO analysis on the top 50 co-expressed genes of CREC family. Fig. S1. PI3K-Akt signaling pathway and protein processing in endoplasmic reticulum of CRC. (A) PI3K-Akt signaling pathway and (B) protein processing in endoplasmic reticulum regulated by CREC family and the top 50 co-expression genes in CRC. [file 12885_2023_11303_MOESM1_ESM.docx]

Supplementary Material

Expression signature and prognostic value of CREC gene family in human colorectal cancer

Junya Ning^1^, Min Liu^1^, Jing Shen^1^, Deping Wang^1^, Lijuan Gao^1*^, Huiyu Li^2, 3*^, Jimin Cao^1*^

^1^ Key Laboratory of Cellular Physiology at Shanxi Medical University, Ministry of Education, and the Department of Physiology, Shanxi Medical University, Taiyuan 030001, China.

^2^ Department of General Surgery, Shanxi Bethune Hospital, Shanxi Academy of Medical Sciences, Tongji Shanxi Hospital, Third Hospital of Shanxi Medical University, Taiyuan 030032, China.

^3^ Tongji Hospital, Tongji Medical College, Huazhong University of Science and Technology, Wuhan 430030, China.

* Correspondence: Jimin Cao, email: [caojimin@sxmu.edu.cn](mailto:caojimin@sxmu.edu.cn)

Huiyu Li, email: [lihuiyu1978@126.com](mailto:lihuiyu1978@126.com)

Lijuan Gao, email: gaolijuan@sxmu.edu.cn

**Table S1.** Characteristics of the patients diagnosed with colorectal cancer

| Term | Value |
| --- | --- |
| Age (years) | 70.5±11.3 (mean ± SD, n=11) |
| Sex: n (%) | Male 7 (64%), Female 4 (36%) |
| Tumor location and subtype (n) | Rectum (2) |
|  | Right half colon (3) |
|  | Left half colon (1) |
|  | Transverse colon (1) |
|  | Ascending colon (1) |
|  | Sigmoid colon (1) |
|  | The rectum and sigmoid colon (1) |
|  | Colic flexure (1) |
| Pathological grading (n) | T4N0M0 (6), T4N2M0 (3), T3N0M0 (1), T3N1M0 (1) |

**Table S2.** Biological processes (BP) of GO analysis on the top 50 co-expressed genes of CREC family

| Term | Description | LogP | Gene Symbols |
| --- | --- | --- | --- |
| GO:0031589 | cell-substrate adhesion | -12.7559 | CALR, CSF1, DMP1, FGA, FGG, FN1, GAS6, CCN1, LAMB1, LAMB2, LAMC1, LGALS1, P4HB, ADAM10, FBN1, SPP1, CDH2, KTN1, APOE, APP, SDC2, IL6, TNC |
| GO:0034975 | protein folding in endoplasmic  reticulum | -8.19596 | CALR, P4HB, DNAJC3, HSP90B1, FN1, NUCB1, SERPINA1, PDIA6, PRKCSH, QSOX1, RCN3, ALB, CALU, ADAM10, CDH2, TNC |
| GO:0060560 | developmental growth involved in morphogenesis | -7.8887 | APOE, APP, CSF1, FN1, TNC, LAMB2, SPP1, AURKA, ADAM10, IGFBP3, IGFBP5, IGFBP7, GAS6, CDH2, SDC2, LGALS1, LAMC1, IL6, PCSK9, CCN1 |
| GO:0007566 | embryo implantation | -7.33041 | CALR, IGFBP7, SPP1, TIMP1, STC2, IGFBP5, GAS6, TNC, IL6, LAMB2, AURKA, LGALS1, CCN1 |
| GO:0001503 | ossification | -7.09621 | AHSG, CSF1, DMP1, TNC, IGFBP3, IGFBP5, CCN1, IL6, SPP1, PRKCSH, AURKA, PCSK9, APOE, LAMB1, LAMC1, APP, IGFBP7, DNAJC3, ADAM10 |
| GO:0042060 | wound healing | -7.09377 | APOE, FGA, FGG, FN1, GAS6, TNC, CCN1, IL6, SERPINA1, TIMP1, CALR, FBN1, P4HB, SPP1, AURKA, AHSG, ALB, APP, CDH2, IGFBP3, PCSK9, LTBP1, RCN3, LGALS1, HRC, IGFBP5, ADAM10, NUCB1, CSF1, STC2, HSP90B1 |
| GO:0030335 | positive regulation of cell  migration | -7.00482 | ADAM10, APP, CALR, CSF1, FN1, GAS6, IGFBP5, CCN1, IL6, LAMB1, SDC2, CDH2, LAMB2, DNAJC3, SDF4, APOE, TNC, HRC, AURKA, LGALS1 |
| GO:0031214 | biomineral tissue development | -6.15181 | AHSG, DMP1, GAS6, CCN1, SPP1, FAM20C, LGALS1, CALR, CSF1, DNAJC3 |
| GO:0042476 | odontogenesis | -5.3688 | CSF1, DMP1, TNC, LAMB1, FAM20C |
| GO:0002274 | myeloid leukocyte activation | -5.30395 | ADAM10, AHSG, APP, CSF1, IL6, SERPINA1, DNAJC3, QSOX1, CKAP4, LGALS1 |

**Table S3.** Cellular components (CC) of GO analysis on the top 50 co-expressed genes of CREC family

| Term | Description | LogP | Gene Symbols |
| --- | --- | --- | --- |
| GO:0005788 | endoplasmic reticulum lumen | -100 | ADAM10, AFP, AHSG, ALB, APOE, APP, CALR, CALU, CDH2, CSF1, DMP1, FBN1, FGA, FGG, FN1, GAS6, HRC, TNC, IGFBP3, IGFBP5, IGFBP7, CCN1, IL6, KTN1, LAMB1, LAMB2, LAMC1, LGALS1, LTBP1, NUCB1, P4HB, SERPINA1, PRKCSH, DNAJC3, QSOX1, RCN1, RCN2, SDC2, SPP1, TIMP1, HSP90B1, STC2, PDIA6, TGOLN2, CKAP4, PRSS23, FSTL1, SCG3, FAM20C, RCN3, VWA1, EVA1A, PCSK9 |
| GO:0062023 | collagen-containing extracellular matrix | -23.7617 | AHSG, APOE, CALR, CDH2, FBN1, FGA, FGG, FN1, TNC, IGFBP7, CCN1, LAMB1, LAMB2, LAMC1, LGALS1, LTBP1, SERPINA1, SDC2, TIMP1, HSP90B1, VWA1, DMP1, ADAM10, APP, GAS6, IL6, QSOX1, SPP1, AURKA |
| GO:0031093 | platelet alpha granule lumen | -16.1116 | AHSG, ALB, APP, FGA, FGG, FN1, GAS6, SERPINA1, QSOX1, TIMP1, SCG3, DNAJC3, ADAM10, CKAP4, SDF4, APOE, CCN1, AURKA, RCN3, IL6, CSF1, SPP1 |
| GO:0033018 | sarcoplasmic reticulum lumen | -8.7883 | CALR, CALU, HRC, HSP90B1, ALB, APOE, FBN1,IL6, LTBP1, PCSK9, APP, TNC, IGFBP5, LGALS1, SPP1, STC2, AHSG |
| GO:0048471 | perinuclear region of cytoplasm | -6.91518 | ADAM10, APP, CALR, CSF1, LAMB1, SPP1, AURKA, HSP90B1, STC2, CKAP4, PCSK9, TIMP1 |
| GO:0043256 | laminin complex | -5.81491 | LAMB1, LAMB2, LAMC1, GAS6, QSOX1, FN1 |

**Table S4.** Molecular Function (MF) of GO analysis on the top 50 co-expressed genes of CREC family

| Term | Description | LogP | Gene Symbols |
| --- | --- | --- | --- |
| GO:0005509 | calcium ion binding | -12.5425 | CALR, CALU, CDH2, DMP1, FBN1, GAS6, HRC, LTBP1, NUCB1, PRKCSH, RCN1, RCN2, HSP90B1, FSTL1, SDF4, RCN3 |
| GO:0005520 | Insulin-like growth factor binding | -6.52558 | IGFBP3, IGFBP5, IGFBP7, CCN1, LTBP1, APOE |
| GO:0008201 | heparin binding | -6.08905 | APOE, APP, FBN1, FN1, CCN1, FSTL1, GAS6, TNC, LTBP1 |
| GO:0030545 | receptor regulator activity | -6.03483 | APP, CSF1, FBN1, GAS6, IL6, SPP1, TIMP1, STC2, PCSK9, ALB, LAMC1, RCN3, IGFBP5, CDH2, FSTL1, CALR |


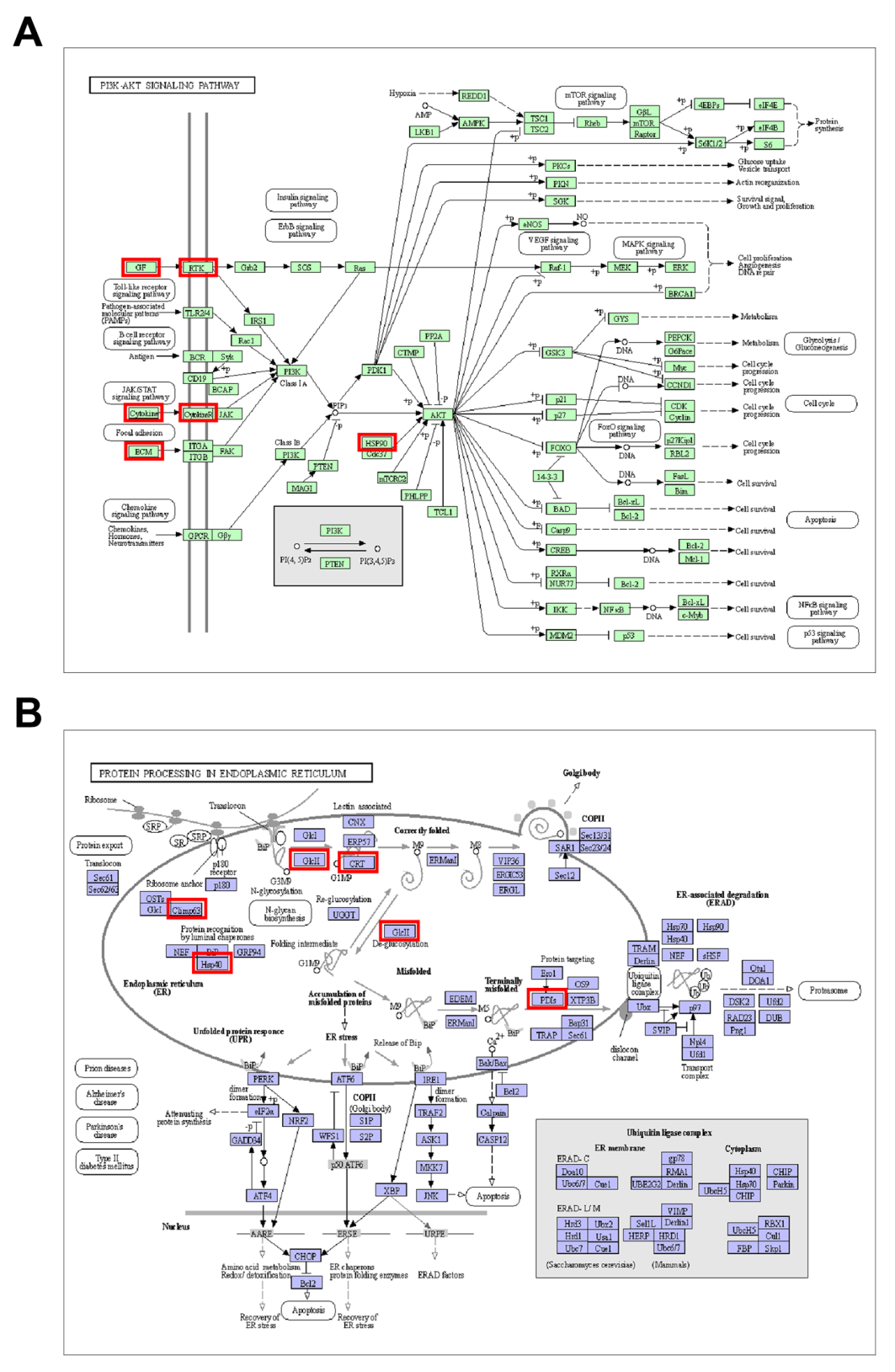


**Fig. S1** PI3K-Akt signaling pathway and protein processing in endoplasmic reticulum of CRC. (A) PI3K-Akt signaling pathway and (B) protein processing in endoplasmic reticulum regulated by CREC family and the top 50 co-expression genes in CRC.
